# Nectin-4 and DNA mismatch repair proteins expression in upper urinary tract urothelial carcinoma (UTUC) as a model for tumor targeting approaches: an ImGO pilot study

**DOI:** 10.1186/s12885-022-09259-z

**Published:** 2022-02-14

**Authors:** Maria Letizia Calandrella, Simona Francesconi, Cecilia Caprera, Claudia Mosillo, Claudia Caserta, Diana Giannarelli, Matteo Corsi, Serena Macrini, Annalisa Guida, Stefano Ascani, Sergio Bracarda

**Affiliations:** 1 Medical and Translational Oncology Unit, Department of Oncology, Azienda Ospedaliera Santa Maria, Terni, Italy; 2 Pathology Unit, Department of Medicine, Medical Clinic Section and Anatomical Pathology, Azienda Ospedaliera Santa Maria, Terni, Italy; 3 Biostatistical Unit, Regina Elena National Cancer Institute, IRCCS, Rome, Italy

**Keywords:** Upper tract urothelial carcinoma, Nectin-4, DNA mismatch repair proteins, MSH2/MSH6 loss, Microsatellite instability

## Abstract

**Background:**

Upper urinary tract urothelial carcinoma (UTUC) accounts for only about 5–10% of all urothelial cancers and is characterized by an aggressive and frequently rapidly fatal behavior. However, detailed knowledge of its molecular profile is still lacking.

**Materials and methods:**

We identified, by chart analysis, patients who underwent radical nephroureterectomy or diagnostic biopsy for UTUC between January 2015 and August 2020 at the Santa Maria Hospital of Terni, in Italy. Eligible patients were required to have also adequate clinical informations and follow-up details. The primary objective of the study was to evaluate DNA mismatch repair (MMR) proteins and Nectin-4 immunohistochemical expression in UTUC, looking also for an eventual correlation between these molecular features. The secondary objective was to investigate genomic instability in the case of a MMR protein loss. Expression of proteins was assessed by using immunohistochemistry and microsatellite instability (MSI) performed by next generation sequencing. Nectin-4 expression was reported using an intensity scoring system (score, 0–3+), instead the expression of DNA MMR proteins was indicated as present (no loss) or not present (loss).

**Results:**

Thirty four cases have been evaluated and 27 considered eligible for the study with their tumor samples analyzed. Nectin-4 was found to be expressed in 44% of cases and 18.5% of patients showed defective-MMR phenotype. We found a significant correlation between Nectin-4 expression and MSH2/MSH6 protein loss. Out of 7 patients with DNA MMR proteins loss or equivocal phenotype, 3 showed MSI.

**Conclusions:**

Our pilot study suggest a possible relationship between Nectin-4 and DNA MMR protein expression in UTUC and a clinically significant correlation between defective MMR phenotype and genomic instability. Because of the possible implications of these data for innovative treatment approaches, the need for further studies in this area is warranted.

**Supplementary Information:**

The online version contains supplementary material available at 10.1186/s12885-022-09259-z.

## Background

Upper urinary tract urothelial carcinoma (UTUC) is a rare and heterogeneous disease, representing about 5–10% of all urothelial tumors with limited evidence in literature but some emerging biologic details [1, 2]. From the clinical point of view, two-thirds of UTUC patients have an invasive disease at diagnosis and 7% of them present metastatic disease [3]. UTUC has an aggressive behavior, with a five-year survival rate of only 60% compared to 77% for all stage urothelial cancer patients [4]. Although similar histological characteristics have been found between UTUC and lower tract carcinoma (LTUC, mainly bladder cancer), increasing evidence suggest UTUC as a distinct biologic disease with specific genetic and epigenetic features [5].

UTUC represents the third most common tumor (5%) in the group of hereditary nonpolyposis colorectal cancer syndrome (HNPCC) related tumors, after colon (63%) and endometrial cancer (9%) [6]. HNPCC, known as Lynch Syndrome, is an autosomal dominant genetic condition associated with germ-line mutations affecting one or several MMR genes (MSH2, MLH1, MSH6, PMS2, EPCAM) with consequent DNA mismatch repair (MMR) enzyme activity lost and increased genomic instability (microsatellite instability - MSI). Of note, while MSI is rare in bladder cancer (3% of cases), a defective DNA MMR system has been documented in more than 15% of sporadic UTUC cases, to reiterate their different genetic profiles [7].

Nectin-4 is a type I transmembrane protein involved in Ca2 + −indipendent cellular adhesion. It is considered a strongly promising biomarker and a specific therapeutic target for urothelial tumors. A moderate to strong staining for Nectin-4 was observed in more than 60% of urothelial cancer and 53% of breast tumor cases, with a consequent convincing rationale for drug development in these tumors [8, 9]. Recent data from phase II and III trials showed a significant efficacy of Enfortumab Vedotin (EV), an antibody-drug conjugate targeting Nectin-4, in patients with urothelial cancers previously treated with platinum and anti-programmed death 1 or anti-programmed death ligand 1 (PD-1/PD-L1) therapies [10, 11].

Aim of this pilot study is to describe the molecular profile of UTUC and investigate eventual correlation between immunohistochemical (IHC) expression of Nectin-4 and DNA mismatch repair (MMR) proteins (MLH1, MHS2, MSH6 and PMS2) in UTUC samples. Moreover, we performed an MSI analysis in patients with MMR-deficient (dMMR) or equivocal phenotype.

## Materials and methods

### Population and tissue samples

We identified, by chart analysis, patients who underwent to a radical nephroureterectomy or diagnostic biopsy for UTUC between January 2015 and August 2020 in our Hospital. Eligible patients were required to have histological samples from their primary tumors or metastatic sites, adequate clinical information, and follow-up details. Appropriate approval was obtained from the local institutional review board and Ethical Committee (CER-Umbria - Protocol Number: 005594, 29 Sept 2020). Written informed consent was obtained from all alive patients.

### IHC analysis of Nectin-4 and MMR proteins expression

Tumor samples were obtained from formalin-fixed and paraffin-embedded surgical specimens. After deparaffinization, rehydration, and the blocking of endogenous peroxidase activity, immunohistochemical staining was performed. For MMR protein expression we used a monoclonal mouse anti–MSH2 antibody (clone G219–1129), a monoclonal rabbit anti–MSH6 antibody (clone SP93), a monoclonal mouse anti–MLH1 antibody (clone M1), and a monoclonal mouse anti–PSM2 antibody (clone A16–4), all using the Ventana Benchmark autostaining system. MMR protein “loss” is defined by the absence of IHC staining in the nucleus of tumor cells. Any nuclear staining was considered as “no loss” of expression.

Regarding Nectin-4 expression we used a rabbit polyclonal antibody (clone ab155692). The intensity of Nectin-4 expression was determined using the histochemical scoring system (H-score) which is defined as the sum of product of the staining intensity (0;1;2;3) and the percentage of stained cells at each intensity level (0–100). The final score ranges from 0 to 300. Finally, the samples were classified as negative (0; H-score, 0–14), low (1+; H-score, 15–99), medium (2+; H-score, 100–199), and high (3+; H-score, 200–300). Microscopic slides from all the specimens were reviewed by a urologic pathologist.

### Molecular analysis of microsatellite instability

Serial 8-μm histological sections of formalin-fixed, paraffin-embedded tissue blocks of normal (N) and tumor (T) were prepared using DNA histology precautions. Normal or tumor tissue was microdissected from unstained slides for each case by overlaying the unstained slide onto the H&E stained side. Dissection of unstained slides was performed in a laminar flow hood after UV irradiation. Only samples with a tumour cell content of at least 25%, which corresponded to the limit of detection determined for the multiplex PCR assay, were included. MSI assays were performed on microdissected DNA, extracted using the “*MagCore Genomic DNA FFPE One-Step*” Kit (*Diatech Pharmacogenetics*). Microvolume samples were quantified by a spectrophotometer system, “*NanoDrop One Microvolume UV-Vis Spectrophotometer* (*Thermo Fisher Scientific*). Paired DNA from normal and tumor tissue specimens were amplified by multiplex amplification PCR (*Titano MSI Kit, Diatech Pharmacogenetics*) with fluorescent primers (Bethesda panel: BAT25, BAT26, D2S123, D17S250, D5S346 and BAT40, D18S58, NR21, NR24, TGFβRII) and subsequent DNA fragment analysis on an automated sequencer. Instability is determined by fragment size analysis on a capillary electrophoresis instrument (*SeqStudio Genetic Analyzer, Thermo Fisher Scientific*) following PCR amplification of DNA from a patient’s normal and tumor tissue samples. The microsatellite status of each sample was determined based on percentage of unstable loci. The status was defined as MSI-high (MSI-H) when 4 or more markers displayed instability and MSI-low (MSI-L) when 1 to 3 markers exhibited instability. A sample was classified as microsatellite stable (MSS) when no MSI was found.

### Statistical analysis

Patient characteristics were reported using descriptive statistics such as mean and standard deviation for quantitative variables and absolute and percentages frequencies for qualitative variables. The association between observed data was evaluated with Fisher exact test. *P*-values < 0.05 were considered statistically significant. The analyses were performed using the statistical software IBM-SPSS v. 21.0.

## Results

### Descriptive analysis

A total of 34 patients were evaluated and 27 were included in the final analysis (3 not available clinical information and 4 not adequate histological samples); the clinicopathological characteristics of patients are summarized in Table 1. Median age of patients at diagnosis was 70 years-old, with a male/female ratio of 8:1. Pyelocaliceal tumors were approximately twice as common as ureteral tumors (ratio 1.5:1). About pathological stage, we have identified mostly pT3 (40.7%) and pNx (92.6%). The 14.8% of patients (4/27) underwent platinum-based adjuvant chemotherapy and 18.5% (5/27) had family history of cancer and second primitive cancers.

**Table 1:** Clinicopathological characteristics of patients

	N (%)
**GENDER**	
M	24 (88.9)
F	3 (11.1)
**AGE (median - range)**	70 (44–86)
**METASTATIC AT DIAGNOSIS**	4 (14.8)
**CONCURRENT BLADDER CANCER**	8 (29.6)
**METACHRONOUS TUMOURS IN LOWER URINARY TRACT**	19 (70.4)
**LOCATION**	
Pelvis	16 (59.3)
Urether	11 (40.7)
**SURGERY**	26 (96.3)
**PATHOLOGICAL T STAGE**	
Ta	1 (3.7)
T1	10 (37.0)
T2	5 (18.5)
T3	11 (40.7)
**PATHOLOGICAL N STAGE**	
N0	1 (3.7)
N1	1 (3.7)
NX	25 (92.6)
**GRADE**	
High	19 (70.4)
Low	8 (29.6)
**LYMPHOVASCULAR INVASION**	
Yes	4 (14.8)
No	23 (85.2)
**HISTOLOGY**	
Urothelial	24 (88.9)
Squamous cell carcinoma	3 (11.1)
**SMOKING**	22 (81.5)
**PRESENCE OF A SECOND PRIMARY CANCER**	5 (18.5)
**FAMILY HISTORY OF CANCER**	5 (18.5)
**PROFESSIONAL RISK FACTOR EXPOSURE**	12 (44.4)
**ADJUVANT CHEMOTHERAPY**	4 (14.8)

### Analysis of IHC features

The overall results are summarized in the Table 2.

**Table 2:** Immunohistochemical features

	N (%)
**NECTIN4**	
NE^a^	1 (3.7)
Negative	14 (51.9)
Low	7 (25.9)
Medium	5 (17.8)
High	0
**MLH1**	
Equivocal	1 (3.7)
Negative	2 (7.4)
Positive	24 (88.9)
**MSH2**	
Equivocal	2 (7.4)
Negative	5 (18.5)
Positive	20 (74.1)
**MSH6**	
Equivocal	1 (3.7)
Negative	6 (22.2)
Positive	20 (74.1)
**PMS2**	
Equivocal	1 (3.7)
Negative	3 (11.1)
Positive	23 (85.2)
**dMMR**	5 (18.5)

^a^Not evaluable

#### Nectin-4

**Fig. 1:**
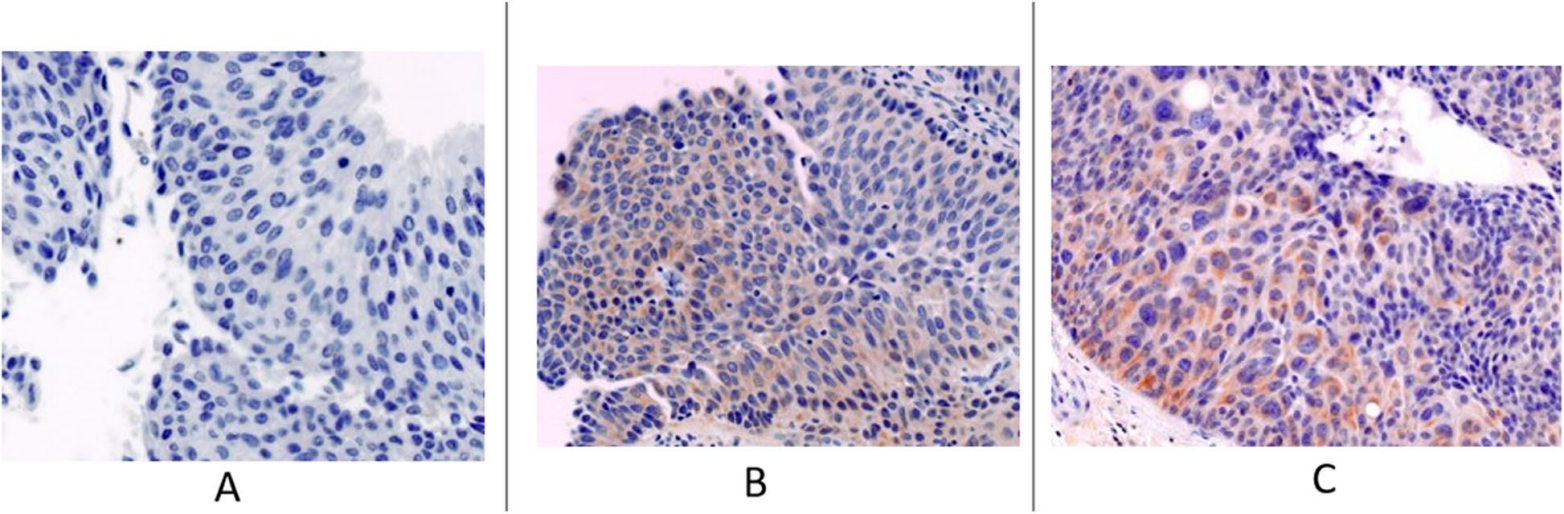
Immunohistochemical expression of Nectin4 in UTUC specimens (**A**) Tissue with negative expression. **B** Tissue with low expression. **C** Tissue with medium expression

The patterns of Nectin-4 IHC expression are shown in Fig. 1.

Nectin-4 was detected in 44.4% of cases (12/27) with a variable expression spectrum (High 0%; Medium 17.8%; Low 25.9%; Negative 51.9%; NV 3.7%).

#### MMR deficiency

**Fig. 2:**
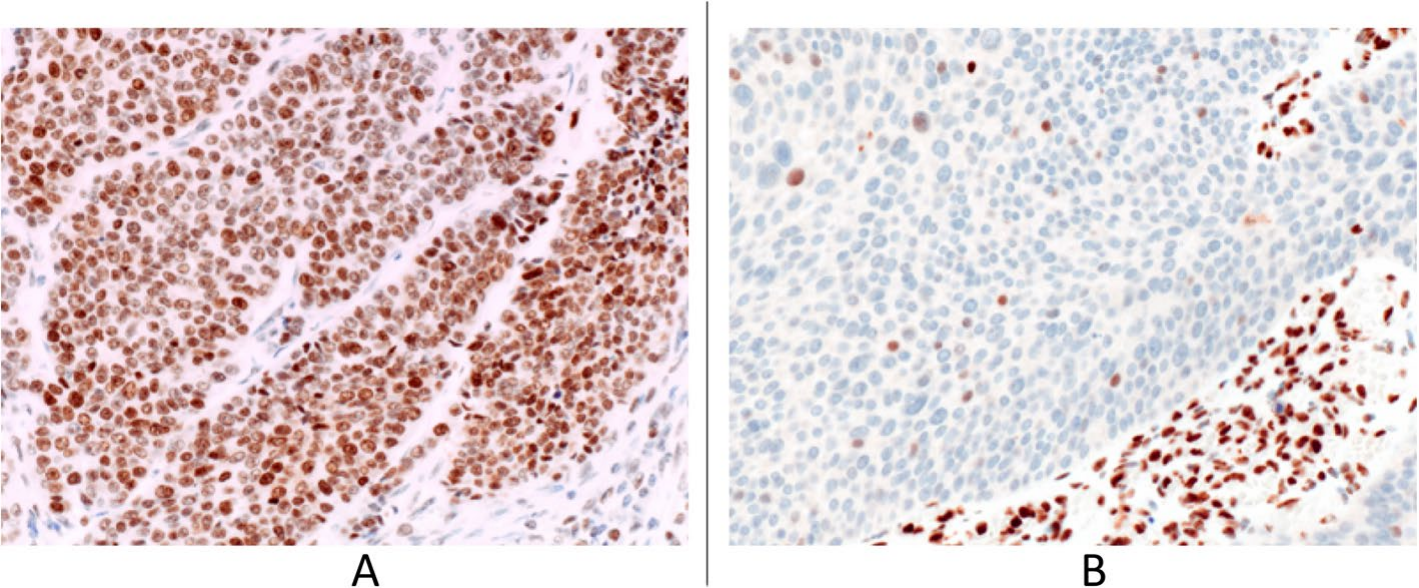
Immunohistochemical expression of MSH6 in UTUC specimens. **A** MSH6 positive with MSS. **B** MSH negative with MSI

The patterns of MMR proteins IHC expression are shown in Fig. 2.

The 18.5% of patients (5/27) showed dMMR phenotype characterized in all cases by loss of MSH2 and MSH6 proteins. Two patients had a “dual lose” profile (MSH2/MSH6 loss): one patient has a loss of expression of 3/4 proteins (MSH2/MSH6/PMS2), and two patients the loss of expression of all 4 proteins (MSH2/MSH6/MLH1/PMS2). In two cases IHC MSH2 expression analysis gave an equivocal result.

#### Cases with expression of Nectin-4 and DNA MMR protein loss

The 5 dMMR cases expressed Nectin-4 with profile of expression Low-Medium and had a pyelocaliceal localization. The median age of this group was 67 years-old (range 57–82); 2/5 had a personal history of second cancers (breast and colon) and 3/5 had a family cancer history (Table 3).

**Table 3:** Clinicopathological characteristics of patients with dMMR profile

CASE	1	2	3	4	5	6	7
**SECOND PRIMITIVE CANCER**	COLON	/	BREAST	/	/	/	/
**FAMILY HISTORY**	DAUGHTER (RECTAL −37 y.o.) MOTHER (RENAL)	FATHER (LUNG) MOTHER(OVARIAN) BROTHER (PANCREATIC 47 y.o.)	/	/	MATHER(ENDOMETRIAL) MATERNAL GRANDFATHER (COLON) MATERNAL AUNT (ENDOMETRIAL) MATERNAL COUSIN (ENDOMETRIAL)	/	/
**HISTOLOGY**	UC^a^	UC	UC	UC	UC	UC	UC
**AGE**	65	62	70	82	57	55	64
**SITE**	PELVIS	PELVIS	PELVIS	PELVIS	URETHER	URETHER	URETHER
**MSS/MSI**	MSS	MSS	MSS	MSS	MSI-H	MSI-L	MSI-L
**NECTIN-4**	MEDIUM	LOW	MEDIUM	LOW	LOW	MEDIUM	NEGATIVE
**MMR lost**	MSH2/MSH6	MSH2/MSH6/PMS2	MLH1/MSH2/MSH6/PMS2	MLH1/MSH2/MSH6/PMS2	MSH2/MSH6	EQUIVOCAL	EQUIVOCAL

^a^Urothelial carcinoma

#### Descriptive analysis of microsatellite

The analysis of microsatellite on DNA extracted from tumor tissue related to cases of dMMR or equivocal phenotype, showed MSI in 3/7 cases: one case MSI-H (8/10 loci) and two cases MSI-L (1/10 e 3/10 loci respectively). The remaining cases analyzed were MSS.

### Statistical correlations

The results of the statistical analysis showed a joint frequency between expression of Nectin-4 and loss of MSH2/MSH6 with statistical significance (*p* = 0.014 and *p* = 0.015 respectively). However, Nectin-4 expression was not significantly associated with MLH1 (*p* = 0.48) and PMS2 (*p* = 0.22). The 60% of patients with MSH2/MSH6 protein loss presented a positive family history for cancer with statistical correlation (*p* = 0.05 and *p* = 0.09 respectively). The loss of the protein MSH2 has been found associated also to female sex (p = 0.09).

## Discussion

Our pilot study reports a clinical and molecular profile of UTUC outlining its own biological identity in respect to LTUC. Regarding clinical features, our data confirmed that about 40% of UTUC patients presented with a locally advanced cancer (pT3) at diagnosis, of note almost all cases had not received a lymphadenectomy (LND) at the time of surgery (pNx 96%), confirming the low trend of this surgical procedure in daily clinical practice, even in the case of bulky primary tumors. Consistently, *Moschini* et al. evaluated a series of 1512 UTUC patients treated with RNU and reported that 64% of cases did not received a concomitant LND, many of them undergoing laparoscopic surgery. In the rare cases in which LND was performed, the procedure was frequently inadequate in terms of number of lymph nodes removed with consequences for optimal staging and cancer specific survival [12, 13]. Moreover, an accurate staging may allow the indication to adjuvant chemotherapy and a more appropriate schedule of follow-up [14].

The expression of Nectin-4, a potential innovative predictive factor, was identified in 44% of cases, mainly represented by low-medium expression levels. The phase II trial EV-201 with Enfortumab vedotin enrolled patients with locally advanced urothelial carcinoma previously treated with PD-1/PD-L1 inhibitor and/or platinum-containing chemotherapy. An overall positive expression of Nectin-4 was shown in 97% of the cases (UTUC and LTUC). Therefore, out of 125 patients, 35% had a UTUC but disjoint IHC data are not available [10]. The analysis of Nectin-4 expression (2394 cases of cancers from 34 different histology), published by *Challita-Eid* and colleagues, showed that 69% of cancer specimens were positive for Nectin-4. In particular, it has been identified in 83% of bladder cancer tissues (High 31% - Moderate 29% - Low 23% - Negative 17%) [8]. However, this evaluation was carried out exclusively on bladder carcinomas. Contrarily, *Tomiyama* et al. showed a Nectin-4 expression in 65/99 (65.7%) of UTUC samples with a predominant low-medium expression profile (1+, 31.3%; 2+, 24.2%; and 3+, 10.1%) [15], confirming, as in our data, a lower expression of Nectin-4 In UTUC in respect to LTUC, and a partially different biological profile.

In the present pilot study, we identified a d-MMR phenotype in 18.5% of the cases. Previous studies have shown a frequency between 4.6 and 27.5% in UTUC populations not otherwise selected [16, 17]. This wide range may depend on the IHC method, the number of samples, and the geographical origin. Moreover, all dMMR cases described were characterized by MSH2/MSH6 proteins loss, outcome consistent with published analysis (70–100%) [18]. Of interest, in the limits of our relatively small sample size, we observed a statistically significant correlation between Nectin-4 expression and MSH2/MSH6 loss (*p* = 0.009 e *p* = 0.005 respectively), indeed all patients with dMMR phenotype showed a Nectin-4 expression.

Pembrolizumab was the first drug to be approved with a tumor-agnostic indication for patients with unresectable or metastatic, MSI-H or dMMR solid tumors [19, 20]. However, in the published trials cases with UTUC were under-represented (and only 2.1% of the cases were urothelial carcinomas). In the phase III study Keynote 045, evaluating pembrolizumab versus second line chemotherapy in recurrent advanced urothelial cancer, UTUC cases represent 14% of the entire population [21]. This study showed a superior survival benefit of pembrolizumab versus the standard of care in all subgroup. Likewise, a case of high-grade UTUC treated with immunotherapy was reported, the patient experienced a partial response (90% shrinkage) after five courses of Pembrolizumab [22]. These experiences suggest a way forward for the use of immune check-point inhibitors in UTUC, but also highlight the importance of a proper patient selection.

Our IHC data about MMR protein expression allowed us to carry out a pre-screening and estimate the prevalence of cases potentially associated with a Lynch syndrome. The median age of the identified 7 patients was 65 years old, compared to 70 years of the study entire population. The median age of onset Lynch-related colon cancer is 45 years-old, about 20–30 years younger in respect to the sporadic forms and it is shown that in patients with Lynch syndrome, UTUC occurs 15 years after other associated oncologic diseases [23, 24]. By using the clinical criteria to screen the risk of a hereditary disease, only 3/7 of patients with dMMR met the classical clinical criteria. Accordingly, many hereditary forms of UTUC may be misclassified as sporadic and their incidence under-estimated with a consequent lack of genetic counseling [25]. Incidence of MSI is about 3% in urothelial bladder but higher in UTUC, up to 15% of the cases [26, 27]. We investigated dMMR phenotype as a surrogate for MSI with a matching rate of 42%. One of the 7 examined resulted MSI-H (8/10 loci) and two cases resulted MSI-L (1/10 and 3/10 loci respectively), and the remaining 4 a MSS phenotype. Data about an eventual correlation rate between the two methods for UTUC are lacking in the literature [28]. However, recent studies showed a rate of concordance of 97.8 and 96.9% for colon and endometrial cancers, respectively [29, 30]. Therefore, more studies with larger samples are needed to assess a possible interchangeability for these data.

The present analysis has several limitations and should be considered only as a preliminary pilot study testing some possible specific details of UTUC cases. First, its retrospective nature, second, the low number of included cases, even considering that UTUC is a rare subpopulation of urothelial cancer. Third limitation point is that data concerning hereditary syndromes should be confirmed by DNA sequencing. Finally, ICH evaluations are operator-dependent and have some analytical variability.

This preliminary experience suggests a way forward for the clinical and molecular characterization of this rare subtype of urothelial cancer to improve knowledge on potential predictive factors and design innovative treatment approaches, also in respect to LTUC.

## Conclusion

Nectin-4 and MMR could be expressed at different levels in UTUC in respect to urothelial bladder cancer. The observed pattern of combination of these data may be tested as a potential predictive tool for the use of a combination of immune- drug conjugates, such as Enfortumab vedotin, and checkpoint inhibitors. The need for further studies in this area is warranted.

## Supplementary Information

**Additional file 1.**

## Data Availability

The datasets used and/or analyzed during the current study are available from the corresponding author on reasonable request.

## Abbreviations

UTUC: Upper tract urothelial carcinoma
MMR: DNA mismatch repair
MSI: Microsatellite instability
LTUC: lower tract urothelial carcinoma
HNPCC: Hereditary nonpolyposis colorectal cancer
EV: ENfortumab-Vedotin
PD1/PDL1: Programmed death 1 or anti-programmed death ligand 1
IHC: Immunohistochemistry
dMMR: Deficient DNA Mismatch Repair
MSI-H: Microsatellite Instability High
MSI-L: Microsatellite Instability Low
MSS: Microsatellite Stability
LND: Lymphadenectomy
