# Supplementary material for: Nectin-4 and DNA mismatch repair proteins expression in upper urinary tract urothelial carcinoma (UTUC) as a model for tumor targeting approaches: an ImGO pilot study
Source: BMC Cancer. 2022 Feb 14;22:168. doi: 10.1186/s12885-022-09259-z (PMC8845253; doi:10.1186/s12885-022-09259-z)
Supplement: Supplementary file 1 — Additional file 1. [file 12885_2022_9259_MOESM1_ESM.zip › contingency tables.docx]

| **nectin4_ * msh6_** | | | | | |
| --- | --- | --- | --- | --- | --- |
| p=0.015 | | | msh6_ | | Totale |
|  |  |  | neg | pos |  |
| nectin4_ | neg | Conteggio | 0 | 13 | 13 |
|  |  | % entro nectin4_ | 0,0% | 100,0% | 100,0% |
|  | pos | Conteggio | 6 | 7 | 13 |
|  |  | % entro nectin4_ | 46,2% | 53,8% | 100,0% |
| Totale | | Conteggio | 6 | 20 | 26 |
|  |  | % entro nectin4_ | 23,1% | 76,9% | 100,0% |

| **nectin4_ * msh2_** | | | | | |
| --- | --- | --- | --- | --- | --- |
| p=0.014 | | | msh2_ | | Totale |
|  |  |  | neg | pos |  |
| nectin4_ | neg | Conteggio | 0 | 13 | 13 |
|  |  | % entro nectin4_ | 0,0% | 100,0% | 100,0% |
|  | pos | Conteggio | 5 | 7 | 12 |
|  |  | % entro nectin4_ | 41,7% | 58,3% | 100,0% |
| Totale | | Conteggio | 5 | 20 | 25 |
|  |  | % entro nectin4_ | 20,0% | 80,0% | 100,0% |

| **sex * msh2_** | | | | | |
| --- | --- | --- | --- | --- | --- |
| p=0.09 | | | msh2_ | | Totale |
|  |  |  | neg | pos |  |
| sex | m | Conteggio | 3 | 19 | 22 |
|  |  | % entro sex | 13,6% | 86,4% | 100,0% |
|  | f | Conteggio | 2 | 1 | 3 |
|  |  | % entro sex | 66,7% | 33,3% | 100,0% |
| Totale | | Conteggio | 5 | 20 | 25 |
|  |  | % entro sex | 20,0% | 80,0% | 100,0% |

| **famil * msh2_** | | | | | |
| --- | --- | --- | --- | --- | --- |
| p=0.05 | | | msh2_ | | Totale |
|  |  |  | neg | pos |  |
| famil | si | Conteggio | 3 | 2 | 5 |
|  |  | % entro famil | 60,0% | 40,0% | 100,0% |
|  | no | Conteggio | 2 | 15 | 17 |
|  |  | % entro famil | 11,8% | 88,2% | 100,0% |
| Totale | | Conteggio | 5 | 17 | 22 |
|  |  | % entro famil | 22,7% | 77,3% | 100,0% |

| **famil * msh6_** | | | | | |
| --- | --- | --- | --- | --- | --- |
| p=0.09 | | | msh6_ | | Totale |
|  |  |  | neg | pos |  |
| famil | si | Conteggio | 3 | 2 | 5 |
|  |  | % entro famil | 60,0% | 40,0% | 100,0% |
|  | no | Conteggio | 3 | 15 | 18 |
|  |  | % entro famil | 16,7% | 83,3% | 100,0% |
| Totale | | Conteggio | 6 | 17 | 23 |
|  |  | % entro famil | 26,1% | 73,9% | 100,0% |

| **nectin4_ * mlh1_** | | | | | |
| --- | --- | --- | --- | --- | --- |
| p=0.48 | | | mlh1_ | | Totale |
|  |  |  | neg | pos |  |
| nectin4_ | neg | Conteggio | 0 | 13 | 13 |
|  |  | % entro nectin4_ | 0,0% | 100,0% | 100,0% |
|  | pos | Conteggio | 2 | 11 | 13 |
|  |  | % entro nectin4_ | 15,4% | 84,6% | 100,0% |
| Totale | | Conteggio | 2 | 24 | 26 |
|  |  | % entro nectin4_ | 7,7% | 92,3% | 100,0% |

| **nectin4_ * pms2_** | | | | | |
| --- | --- | --- | --- | --- | --- |
| p=0.22 | | | pms2_ | | Totale |
|  |  |  | neg | pos |  |
| nectin4_ | neg | Conteggio | 0 | 13 | 13 |
|  |  | % entro nectin4_ | 0,0% | 100,0% | 100,0% |
|  | pos | Conteggio | 3 | 10 | 13 |
|  |  | % entro nectin4_ | 23,1% | 76,9% | 100,0% |
| Totale | | Conteggio | 3 | 23 | 26 |
|  |  | % entro nectin4_ | 11,5% | 88,5% | 100,0% |
