# Supplementary material for: Nectin-4 and DNA mismatch repair proteins expression in upper urinary tract urothelial carcinoma (UTUC) as a model for tumor targeting approaches: an ImGO pilot study
Source: BMC Cancer. 2022 Feb 14;22:168. doi: 10.1186/s12885-022-09259-z (PMC8845253; doi:10.1186/s12885-022-09259-z)
Supplement: Supplementary file 1 — Additional file 1. [file 12885_2022_9259_MOESM1_ESM.zip › SUPPLEMENTARY FILES LEGEND.docx]

SUPPLEMENTARY FILES

N1. UTUC DATABASE: file-excel which shows the clinicopathological and IHC features

N2. CONTINGENCY TABLES: two-way frequency table as results of statistical analysis
